# Assessing Cardiomyocyte Excitation-Contraction Coupling Site Detection From Live Cell Imaging Using a Structurally-Realistic Computational Model of Calcium Release

**DOI:** 10.3389/fphys.2019.01263

**Published:** 2019-10-02

**Authors:** David Ladd, Agnė Tilūnaitė, H. Llewelyn Roderick, Christian Soeller, Edmund J. Crampin, Vijay Rajagopal

**Affiliations:** ^1^Systems Biology Lab, Department of Biomedical Engineering, School of Mathematics and Statistics, University of Melbourne, Melbourne, VIC, Australia; ^2^ARC Centre of Excellence in Convergent Bio-Nano Science and Technology, School of Chemical and Biomedical Engineering, University of Melbourne, Melbourne, VIC, Australia; ^3^Cell Structure and Mechanobiology Group, Department of Biomedical Engineering, University of Melbourne, Melbourne, VIC, Australia; ^4^Laboratory of Experimental Cardiology, Department of Cardiovascular Sciences, KU Leuven, Leuven, Belgium; ^5^Living Systems Institute, University of Exeter, Exeter, United Kingdom

**Keywords:** cardiomyocyte, calcium signaling, excitation-contraction coupling, cellular cardiac physiology, ryanodine receptor, live cell imaging, computational model, validation

## Abstract

Calcium signaling plays a pivotal role in cardiomyocytes, coupling electrical excitation to mechanical contraction of the heart. Determining locations of active calcium release sites, and how their recruitment changes in response to stimuli and in disease states is therefore of central interest in cardiac physiology. Current algorithms for detecting release sites from live cell imaging data are however not easily validated against a known “ground truth,” which makes interpretation of the output of such algorithms, in particular the degree of confidence in site detection, a challenging task. Computational models are capable of integrating findings from multiple sources into a consistent, predictive framework. In cellular physiology, such models have the potential to reveal structure and function beyond the temporal and spatial resolution limitations of individual experimental measurements. Here, we create a spatially detailed computational model of calcium release in an eight sarcomere section of a ventricular cardiomyocyte, using electron tomography reconstruction of cardiac ultrastructure and confocal imaging of protein localization. This provides a high-resolution model of calcium diffusion from intracellular stores, which can be used as a platform to simulate confocal fluorescence imaging in the context of known ground truth structures from the higher resolution model. We use this capability to evaluate the performance of a recently proposed method for detecting the functional response of calcium release sites in live cells. Model permutations reveal how calcium release site density and mitochondria acting as diffusion barriers impact the detection performance of the algorithm. We demonstrate that site density has the greatest impact on detection precision and recall, in particular affecting the effective detectable depth of sites in confocal data. Our findings provide guidance on how such detection algorithms may best be applied to experimental data and give insights into limitations when using two-dimensional microscopy images to analyse three-dimensional cellular structures.

## 1. Introduction

Each heartbeat is induced by an efflux of calcium (Ca^2+^) from the sarcoplasmic reticulum (SR) into the cytosol. This release of Ca^2+^ through clusters of ryanodine receptors (RyRs) raises the bulk cytosolic Ca^2+^ concentration from 0.1 μM to ≈1 μM within 30 ms and results in exposure of cross-bridge binding sites on the actin filaments, facilitating cellular contraction (Bers, 2002; Gilbert et al., 2019). Confocal (Soeller and Cannell, 2002) and super-resolution (Hou et al., 2015) microscopy imaging of immuno-labeled cardiac tissue preparations have previously revealed the spatial organization of RyR clusters but measurements from fixed tissues are limited in their ability to provide insights into function.

Functional imaging of Ca^2+^ release in cardiomyocytes is possible using fluorescence-labeled confocal microscopy. Unfortunately, these images suffer from low signal-to-noise ratio and their two-dimensional nature collapses the dynamic three-dimensional system. Recently, Tian et al. (2017) proposed an adaption of the CLEAN family of methods from radio astronomy signal analysis (Högbom, 1974) to detect Ca^2+^ release sites in live cardiomyocytes. This approach detects RyR clusters as point sources and iteratively deconvolves them from the signal with point spread functions to determine cluster locations in confocal fluorescence images. Using this algorithm (CaCLEAN), the authors demonstrated the possibility of detecting the time-dependent functional response of RyR clusters in live cell preparations using widely available experimental methods.

However, questions remain regarding the suitability and performance of this proposed approach. Foremost is the inability to define ground truth locations of active RyR clusters to compare against those detected when applying the method to experimental data. Further complicating detection is that, compared to the near-vacuum between astronomical objects, the diffusive volume between clusters of RyRs in cardiomyocytes is heterogeneous, consisting primarily of myofibrils and mitochondria. This issue is relevant to the local distribution of diffusing Ca^2+^: while homologs of cell membrane Ca^2+^ transport channels exist on mitochondria and exhibit a modest buffering effect, Ca^2+^ flux between the cytosol and intra-mitochondrial space is negligible compared to other cytosolic Ca^2+^ pathways under normal physiological conditions (Williams et al., 2013). Given this potential barrier-like effect of mitochondria to diffusing Ca^2+^, we hypothesized that Ca^2+^ reflecting against mitochondria could result in additional false positive detection events. Finally, the spatial distribution of Ca^2+^ sources diffusing into the image impacts detection performance. High densities of release sites saturate the two-dimensional imaging space more readily and at shorter distances from the acquisition plane in comparison to lower site densities. Typical experimental preparations of cardiomyocytes are imaged in a longitudinal orientation that captures clusters spaced across sarcomeres (≈2μm apart) and within z-disk populations, where nearest-neighbor cluster distances in rat ventricular cardiomyocytes are ≈0.66μm (Soeller et al., 2007).

**Figure 1:**
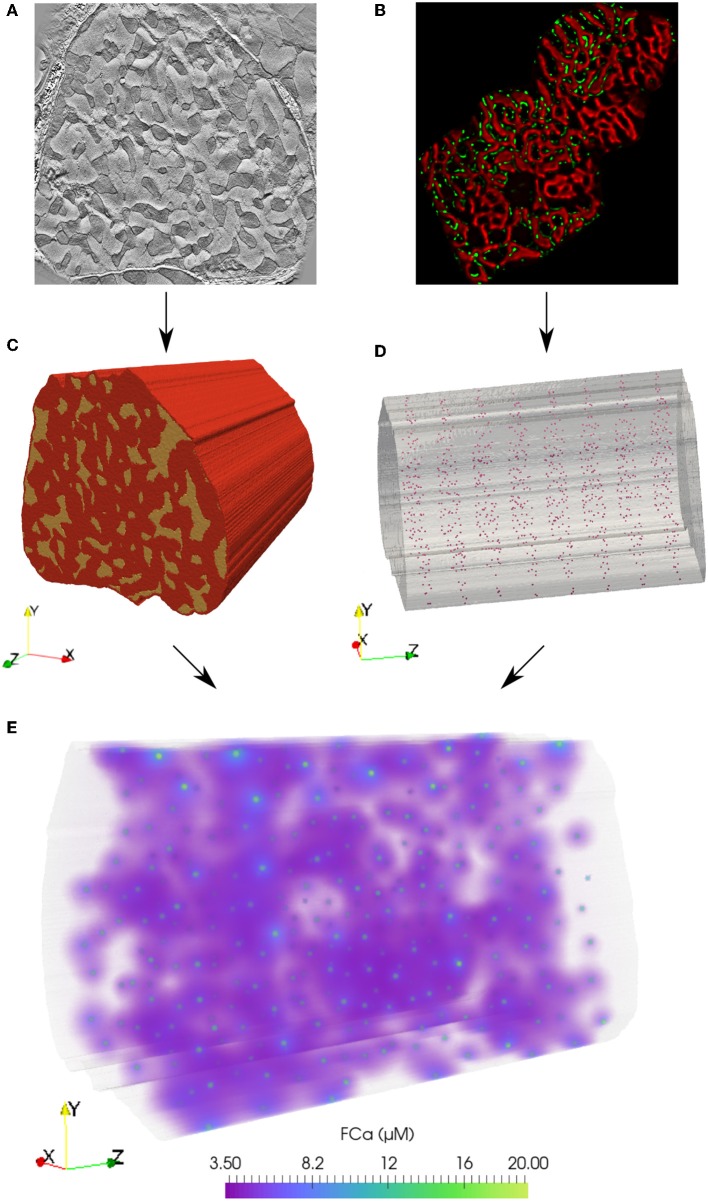
Finite element model of Ca^2+^ reaction-diffusion in an eight sarcomere section of a cardiomyocyte. **(A)** From an electron tomography imaging stack, myofibril and mitochondria regions were segmented from a slice at the depth of a z-disk. **(C)** This geometry was extruded 16 μm (in the direction shown as z here) to create a three-dimensional eight-sarcomere model. Mitochondrial regions shown in yellow; red volume indicates the myofibrillar and cytosolic region. **(B)** Statistical analysis of immuno-labeled microscopy data (RyR clusters shown in green) was used to determine inter-cluster spacing distributions. **(D)** RyR cluster locations in the model were defined at mitochondrial and myofibrillar border regions based on statistical spacing distributions. **(E)** A reaction-diffusion finite element model simulates the release of Ca^2+^ from the RyR clusters during the rising phase (first 30 ms) of the Ca^2+^ transient. Volume rendering of the fluorescence-bound Ca^2+^ (FCa) field shown at *t* = 15 ms.

To address these issues, we develop a spatially detailed computational model of an eight sarcomere section of a cardiomyocyte by extruding an electron tomography image. Within this three dimensional domain, we simulate reaction-diffusion of Ca^2+^ emanating from RyR clusters. Our model captures these mechanics during the rising phase of the Ca^2+^ transient- the first 30 ms following the membrane depolarization before contraction begins- upon which CaCLEAN detection operates (Tian et al., 2017). This model builds on previous work (Rajagopal et al., 2015), which introduced the approach of computationally fusing organelle structure from electron tomography imaging (see Figure 1A) with distributions of RyR clusters from statistical analysis of immuno-labeled confocal microscopy protein localization data (see Figure 1B). The present model extends this approach to elucidate the complex environment of Ca^2+^ signals emitted from release sites at the z-disks and merging at the m-lines that is responsible for coordinated cellular contraction.

The results of this model are subsequently used to simulate confocal fluorescence images. These images are then analyzed with CaCLEAN to assess RyR cluster detection performance in the context of known ground truth locations to quantify true positives (hits), false negatives (misses), and false positives (algorithmic artifacts). Our findings indicate that the presence of mitochondria only has a marginal negative impact on detection at a typical experimental imaging resolution. We quantify the impact of inter-cluster spacing on detection performance, as well as how far from the imaging plane clusters are most accurately detected. We estimate the recall and precision of the algorithm as between 69–82%, depending on the density of cluster locations and their distance from the imaging plane. Our analysis therefore serves as a reference for future applications or extensions of CaCLEAN and similar release-site detection algorithms, providing quantitative analysis of performance using a physics-based modeling framework with known ground truths.

## 2. Results

### 2.1. Simulating Microscopy Data Allows for Assessment of Detection Performance With Known Ground Truth Values

A spatially detailed finite element (FE) computational model of an eight sarcomere section of a cardiomyocyte was constructed to evaluate RyR cluster detection. The algorithms used to generate the model (Rajagopal et al., 2015) provided unique RyR cluster distributions at each z-disk (see Figure 1D). The influence of mitochondria acting as diffusion barriers and the spatial arrangement of RyR clusters on the performance of CaCLEAN were studied using these models. This resulted in four model permutations:

1. CASE 1 (high cluster density, no mitochondria): A case with high cluster density (*N* = 984, 123 clusters per z-disk) based on statistical analysis of nearest neighbor distributions of clusters from immuno-labeled confocal microscopy data (Figure 1B). The cell volume was treated as a homogeneously diffusive continuum.
2. CASE 2 (high cluster density, with mitochondria): Mitochondrial regions (Figure 1C) were segmented from the modeled cell volume, creating boundaries acting as obstacles to diffusion. RyR cluster distributions were defined as in case 1.
3. CASE 3 (low cluster density, no mitochondria): A case with a relatively low cluster density (*N* = 408 or 51 per z-disk) and an additional constraint of a minimum spacing of 1 μm spacing between cluster centers. The cell volume was treated as a homogeneously diffusive continuum.
4. CASE 4 (low cluster density, with mitochondria): Mitochondrial obstacles were included as in case 2; RyR cluster distributions were defined as in case 3.

**Figure 2:**
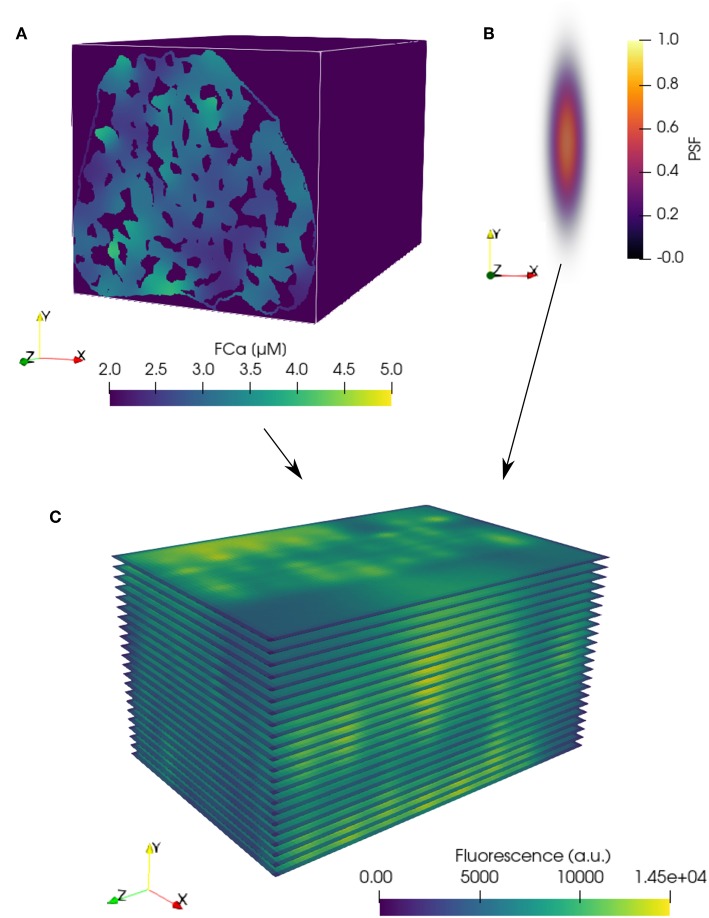
Simulation of confocal fluorescence microscopy images from FE model results. **(A)** FE FCa field data (see Figure 1E) interpolated onto a regular grid with 53.75 nm resolution in each coordinate direction. Three-dimensional convolution of the interpolated FCa data with a **(B)** point spread function (PSF) produces blurring typical of confocal fluorescence microscopy data. **(C)** Blurred data resampled at a pixel resolution of 215 nm within the 22 simulated two-dimensional imaging planes in the volume. **(A,C)** are shown at a single time point, *t* = 15 ms.

In the three-dimensional FE model, the fluorescence-bound Ca^2+^ (FCa) field was calculated based on the time-dependent reaction-diffusion emanating from the surrounding cluster sources (see Figure 1E). The FCa field was next interpolated onto a regular grid (see Figure 2A) and temporally downsampled to 5 ms intervals. The FCa field was then convolved in three dimensions with a point spread function (PSF, Figure 2B) to simulate optical blurring and light noise (SNR = 100) was added. This dataset was then resampled at 215 nm pixel resolution in two dimensions at 22 equidistant slices as indicated in Figure 2C to obtain simulated images that mimic fast 2D confocal images obtained in typical experiments.

**Figure 3:**
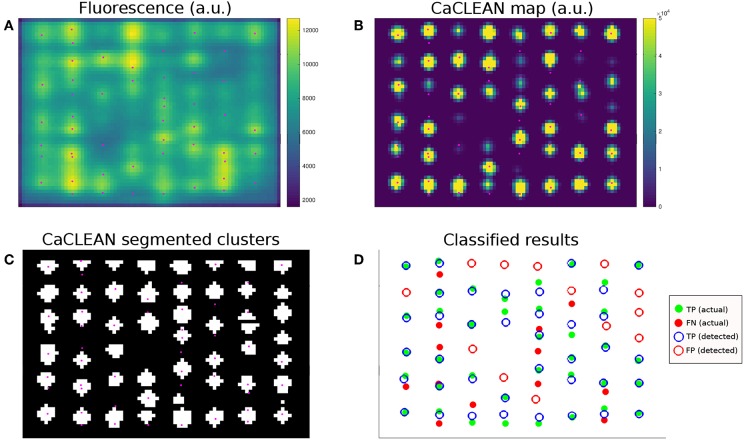
Example of CaCLEAN detection and classification against modeled locations. **(A)** Simulated confocal fluorescence microscopy image at *t* = 15 ms for the densely packed cluster case (*N* = 123 per z-disk) with mitochondria. This represents one slice from the middle of the stack shown in Figure 2C. Modeled RyR cluster center locations within 280 nm of the imaging plane shown in magenta (also in **B,C**). **(B)** CaCLEAN map of the fluorescence signal. **(C)** Segmented clusters detected by CaCLEAN. **(D)** Statistical classification of detected cluster locations vs. actual (ground truth) modeled locations. Modeled release sites are represented by filled dots and detected sites are open circles. A correct detection is therefore represented by a green dot surrounded by a blue circle, while false positives (detection errors) are red circles and false negatives (missed modeled locations) are red dots.

**Figure 4:**
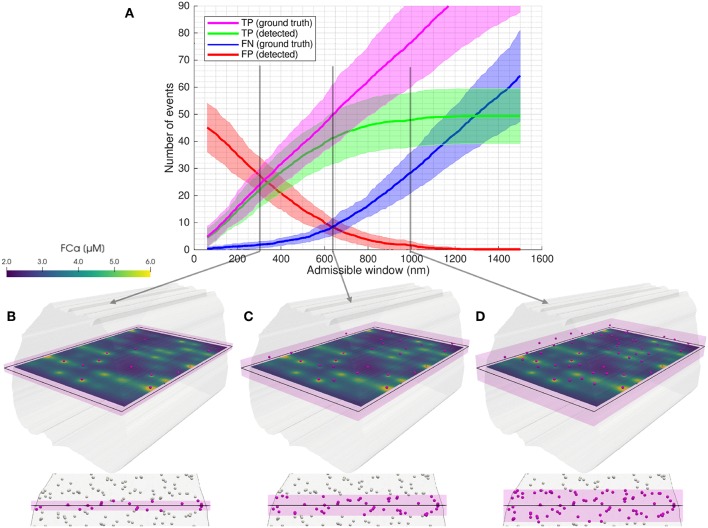
Illustration of the admissible window parameter. The admissible window defines the distance tolerance from the simulated imaging plane for RyR cluster centers to be considered ground true positives. **(A)** Statistical classifier CaCLEAN results as a function of the admissible window for low cluster density, no mitochondrial barriers (case 3). Twenty two images were simulated with equidistant spacing through the model volume. Solid lines represent mean values and shaded regions indicate one standard deviation of values. Figure S1 shows similar results across the four model permutations. In **(B–D)** an example image slice is shown, with clusters considered TP (ground truth) for admissible windows of **(B)** 300 nm, **(C)** 640 nm, and **(D)** 1,000 nm in magenta. An oblique view is shown above, along with FCa model results at *t* = 15 ms. Below, an axial view perpendicular to the imaging plane is shown (looking through the modeled volume, with clusters outside the admissible window shown in white). The admissible window is indicated in pink shading.

The resulting time-dependent, simulated confocal fluorescence microscopy images at each slice (Figure 3A) were then analyzed with CaCLEAN to produce maps (Figure 3B), which were then segmented into individual clusters (Figure 3C). To measure algorithm performance we conducted statistical classification (Figure 3D) using the modeled locations as the actual class (ground truths) and the detected locations as the predicted class to identify true positives (TP), false negatives (FN), and false positives (FP). TP (ground truth) represented the modeled clusters in a given admissible window (see below and Figure 4). TP (detected) represented those TP (ground truth) correctly detected by CaCLEAN. FN identified the TP (ground truth) “missed” by CaCLEAN TP (detected). FP represented the cluster locations detected by CaCLEAN that were not consistent with TP (ground truth) locations.

### 2.2. Cluster Distance From the Imaging Plane Reveals the Trade-Off Between Recall and Precision

For each simulated imaging plane, modeled RyR clusters were considered TP (ground truth) if their centers were within a distance threshold from the imaging plane we referred to as the “admissible window” (see Figure 4). As illustrated in Figure 4A, the number of TP (ground truth, magenta) increased linearly with the admissible window as the window incorporated more of the modeled cluster locations. The number of TP (detected, green) events approached a limit as the collapsed two-dimensional imaging space became saturated with available TP (ground truth) locations and the signal diffusing from far-field clusters did not reach the image space.

Clusters located very near the z-depth^1^ of the imaging plane were the most likely to affect the signal and be detected by the algorithm, as indicated by the consistently low FN (blue) at low admissible window. However, with a very narrow admissible window, FP (red) were prevalent since clusters located just outside of this arbitrary tolerance still diffused into the imaged space and were detected by CaCLEAN. Conversely, with a widening definition of the admissible window, FP dropped and FN increased due to the asymptotic behavior of TP (detected).

**Figure 5:**
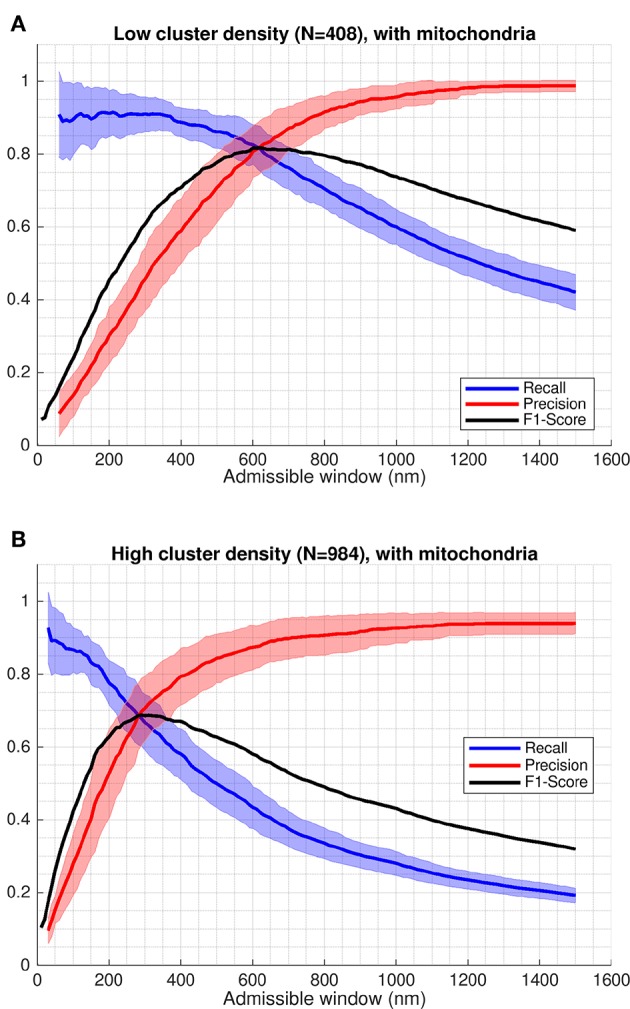
Recall and precision of CaCLEAN applied to simulated microscopy data. Recall, precision and f1-score evaluated based on classification results. Shaded regions indicate one standard deviation of recall and precision values. The f1-score shown is evaluated based on the mean values for precision and recall (solid red and blue lines). In both cases shown, regions representing mitochondria act as barriers to diffusion. **(A)** A minimum spacing of 1 μm is enforced between modeled clusters of RyRs. **(B)** Cluster spacing is determined by statistical analysis of RyR cluster distributions from immuno-labeled super-resolution microscopy data.

We quantified algorithmic performance using well-established measures to capture the impact of false positives and false negatives on performance: recall, precision, and f1-score (Nisbet et al., 2018; Berrar, 2019). These metrics were evaluated for the four model permutations using classifiers as a function of the admissible window (see Figure 5, Figure S2, Table 1). Recall, as the ratio of TP (detected) to TP (ground truth), provided fractional measure of correctly identified clusters. Precision, as the ratio of the number of TP (detected) to all detected sites (TP and FP), provided the fraction of detected clusters that were not FP. F1-score represented the harmonic mean of precision and recall values. Higher values indicated better performance in all three measures.

**Table 1:** Ca^2+^ release site detection performance results at maximum f1-score in each model.

**Model**	**F1-Score**	**Recall**	**Precision**	**Admissible window (nm)**
Low RyR density, no mitochondria	0.83	0.83 ± 0.06	0.83 ± 0.08	630
Low RyR density, with mitochondria	0.82	0.82 ± 0.06	0.82 ± 0.07	620
High RyR density, no mitochondria	0.70	0.67 ± 0.08	0.74 ± 0.10	290
High RyR density, with mitochondria	0.69	0.68 ± 0.07	0.70 ± 0.09	290

### 2.3. Inter-cluster Spacing Has a Greater Impact on CaCLEAN Performance Than Mitochondrial Diffusion Barriers

Whereas there may be applications where optimizing for recall or precision may be more appropriate, we used the f1-score as a single general measure, equally weighting the influence of misses and false positives in interpretation of performance. Peak values of the f1-score in the cases including mitochondria barriers ranged between 0.69 at an admissible window of 620 nm in the high cluster density case and 0.82 at an admissible window of 290 nm in the low cluster density case (see Figure 5 and values reported in Table 1). Notably, the maximum f1-score values corresponded closely with the precision-recall break-even point (the intersection of the recall and precision curves), as seen in Figure 5 or by comparing values reported in Table 1 vs. Table 2. This point identifies where the number of false positives is nearest to the number of false negatives (Christen and Goiser, 2007), thus also predicting approximately the correct number of clusters overall. Curve fits for mean precision and recall values are also provided in the Supplementary Material (see Figure S3 and Tables S1, S2).

**Table 2:** Ca^2+^ release site detection performance results at the precision-recall break-even point, where Precision ≈ Recall and the number of false positives ≈ the number of false negatives.

**Model**	**F1-Score**	**Recall**	**Precision**	**Admissible window (nm)**
Low RyR density, no mitochondria	0.83	0.83 ± 0.06	0.83 ± 0.08	630
Low RyR density, with mitochondria	0.82	0.82 ± 0.06	0.82 ± 0.07	620
High RyR density, no mitochondria	0.69	0.69 ± 0.08	0.69 ± 0.12	270
High RyR density, with mitochondria	0.69	0.69 ± 0.08	0.68 ± 0.09	280

**Figure 6:**
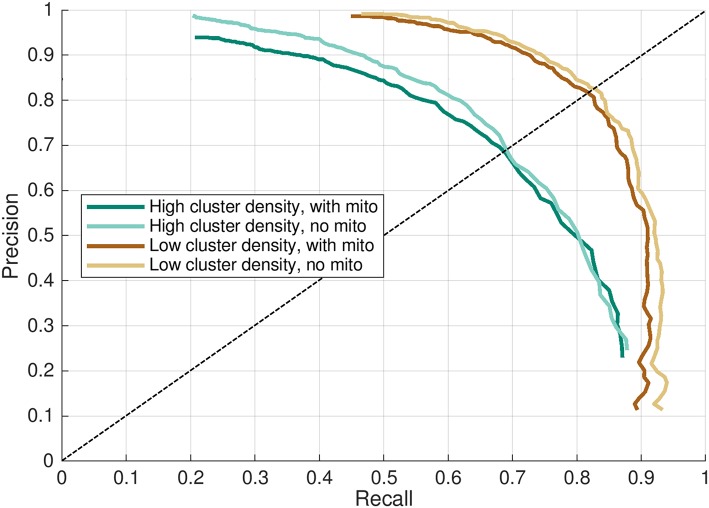
Precision-recall curve for CaCLEAN in four models. Recall and precision mean values from results shown in Figure 5 plotted as a precision-recall curve. Higher values indicate better performance in each axis. Dashed line indicates the precision-recall break-even point, where recall = precision.

The effect of mitochondria acting as barriers to diffusion had a slight consistently negative impact on the evaluated performance when compared against cases where these regions were modeled as diffusing homogeneously with the cytosol. This effect was most evident in the precision-recall curves in Figure 6, where “with mitochondria” model permutations show only slightly lower performance in both axes than the “no mitochondria” alternatives. This was also visible in the peak f1-score and precision-recall break-even values reported in Tables 1, 2 and by close visual inspection of Figure S2. One point of difference also observable in Figure S2 is that precision appeared to asymptotically approach 1 in all cases except the densely-packed clusters with mitochondria case (case 2), where additional false positive events resulted in a slight drop of the maximum far-field precision to 0.95.

**Figure 7:**
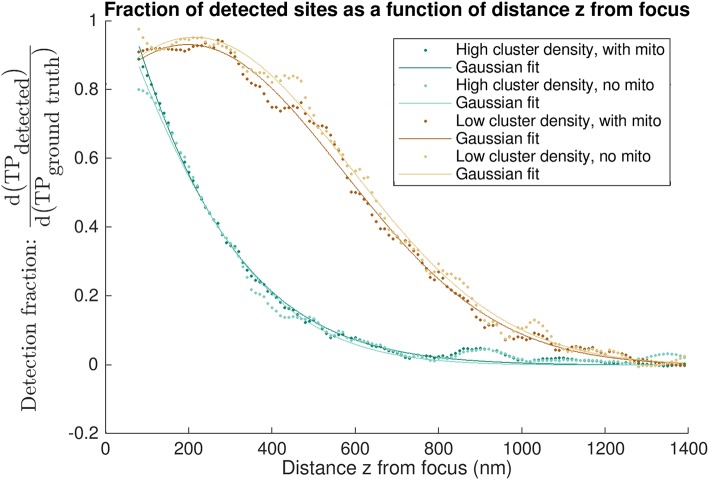
“Differential recall”: axial dependence of detection of true positive sites. Each curve shows the fraction of detected sites as a function of the distance z from the nominal focal plane. Differential recall at distance z is the fraction of all detected to modeled sites within 5 nm of that z depth i.e., within a 10 nm band centered about the distance z above and below the focal plane. A value of 1 is equivalent to the detection of all sites at a given z-depth. The various curves are calculated for different models, having a high or low density of sites and either using homogeneous diffusion (“no mito”) or obstacles to diffusion wherever mitochondria are (“with mito”). Data points shown are the result of applying a smoothing filter (see Figure S5 for the low density, no mito case shown in light brown). Solid lines indicate single-term Gaussian fits to the filtered data.

Finally, we evaluated the fraction of clusters detected by CaCLEAN as a function of z-distance from the focal imaging plane to identify how detection of individual clusters decayed with increasing distance from the imaging plane (see Figure 7). This analysis again highlighted the effect of inter-cluster spacing: when using CaCLEAN to detect sites in models with a high density of release sites the effective z-response of detection falls much more steeply than when a low density of true release sites was simulated.

## 3. Discussion

We developed a computational model of the complex environment of Ca^2+^ diffusing into the intracellular space of a cardiomyocyte. Processing the reaction-diffusion model results to simulate confocal fluorescence microscopy data allowed for quantitative assessment of the performance of detection of Ca^2+^ release sites against known ground truth values in the context of realistic cellular physics. Statistical classification identified true positives, false positives, and false negatives^2^; enabling analysis in terms of recall (sensitivity, hit rate, or true positive rate), precision (positive predictive value), and f1-score (the harmonic mean of precision and recall).

### 3.1. Release Site Detection Performance Is Dependent on Distance From the Image Plane

A key variable in the performance analysis was the definition of which modeled clusters were considered ground truths in the statistical classification at each imaging plane. We introduced the “admissible window” variable for admitting clusters as ground truth values based on the through-imaging-plane distance of cluster centers, as illustrated in Figure 4. Parameterizing algorithmic performance in terms of the admissible window provided a more complete picture of which clusters were being detected and highlighted the inherent trade-off between precision and recall.

The admissible window is also of practical interest to those seeking to use CaCLEAN (or another detection algorithm) on their experimental data. This parameter can be used as an indicator of the maximum relevant depth of clusters detected by the algorithm in the image. As evident in Figure 5, interpretation of release site distance from the imaging plane is strongly dependent on a user's performance requirements and the density of clusters in the sample. A user seeking to determine maximum relevant depth should first determine whether they are more willing to sacrifice precision or recall, deciding whether misses or false positives are of greater concern in their application. In applications where these factors are of equal importance, f1-score values give a performance measure combining recall and precision. In this case peak f1-score values closely correspond with the precision-recall break-even point, where the number of false positives is equal to the number of false negatives (thus still detecting approximately the correct number of clusters).

For example, if a user is confident that clusters in a sample are likely at least 1 μm apart and is satisfied with 80% precision and recall, the maximal relevant depth for clusters diffusing into the image would be ≈620 nm based on Figure 5A and Table 1. From the recall curve, the same user may also be interested to find the algorithm will likely correctly identify 90% of all clusters within 350 nm of the imaging plane. The recall curves in Figure 5 therefore also communicate how the detection performance for an active cluster population drops as further away clusters are considered detectable.

Analysis of performance in terms of the admissible window provides a basis for assessing “cumulative recall,” i.e., the detection of all clusters within a given tolerance of the focal plane. We also evaluate “differential recall” in Figure 7, i.e., the detection fraction of clusters at distance z from the imaging plane. This communicates how the detection fraction of individual clusters drops with distance from the focal plane and emphasizes the importance of cluster density: detection falls much more steeply in the high density cases. In other words, depending on how many sites are active the z-response of detection changes. In experiments, this means that in cases where all sites release, e.g., after stimulation with a β-adrenergic agonist, the detected sites are on average from regions closer to the focus than when recording in conditions of partial block where fewer sites are available.

#### 3.1.1. The Optical PSF Does Not Determine Detection Depth

The shape of the point spread function (PSF, see Figure 2B) weights the interpolation and blurring of the three-dimensional data into a two-dimensional image at each time step. Choice of PSF can impact the fidelity of the data interpolation into the image and, subsequently, detection performed on the interpolated data. However, it should be noted that the optical depth of the PSF does not directly influence how far from the image clusters may be located or detected. Site detection operates on the fluorescence signal emitted by Ca^2+^ released from RyR clusters, rather than directly capturing these sites in the optical depth of the microscope. Figure S4 shows the recall, precision, and f1-score values using PSFs with half and double the full width at half maximum (FWHM) dimensions of the baseline PSF settings. The tighter half-FWHM PSF case produces less blurring and slightly improves recall, while the broader double-FWHM PSF case produces more blurring to reduce FP events (likely due to mitochondria) and slightly improves precision.

### 3.2. Detection Performance Is Inversely Related to Release Site Density

In addition to distance from the imaging plane, further factors complicate whether the signal from a given cluster will reach the imaging plane and whether it is detectable in both our model and actual experimental data. Individual cluster firing time and strength variability biases detection of early and stronger events. Some false positives are produced as algorithmic artifacts. Proximity to other clusters can cause signals to merge or cover each-other- especially with increased cluster density. For instance, two separate clusters the same z-distance above and below the imaging plane but at the same x-y location in the imaging plane can only register as a single site in the imaged space. This identifies an inherent drawback resulting from using two-dimensional images as a basis for describing a three-dimensional system.

#### 3.2.1. Determining Physiologically Representative Cluster Distributions

RyR cluster distribution spacing varies across species, with nearest-neighbor spacings reported as 0.66 ± 0.06 μm in rat and 0.78 ± 0.07 μm in human (Soeller et al., 2007). Clusters located in the periphery of mouse myocytes are more irregularly spaced than those located in the cell interior (Hiess et al., 2018). These distributions also alter during development, with RyR clusters in rabbits changing from majority peripheral clusters with ≈0.7 μm spacing in neonates to majority interior clusters with ≈2 μm regular spacings between z-disks (Dan et al., 2007). In addition to spatial locations of RyR protein clusters, another consideration is functional response of clusters- an area where CaCLEAN shows unique promise. Tian et al. (2017) used the algorithm to explore how firing reliability decreases with increased stimulation frequency and increases under beta-adrenergic stimulation.

We investigated the impact of cluster spacing on detection performance by analysing two cluster distribution types. In the “high cluster density” cases, cluster locations at each z-disk were defined based on statistical analysis of cluster distributions from immuno-labeled confocal images and applied to admissible locations bordering mitochondria and myofibrils segmented from electron tomography data (Rajagopal et al., 2015). This simulated scenarios where all RyR clusters identifiable by immuno-labeling^3^ fired during the 30 ms period modeled. This was considered a reasonable upper bound on cluster distribution density since it is unlikely that all clusters would fire for a single excitation cycle under normal physiological conditions. Tian et al. (2017) used CaCLEAN to estimate cluster re-fire rates of ≈ 62.8% in mouse atrial myocytes, with only ≈10% of clusters always firing. However, the authors also showed that β-adrenergic stimulation can increase recruitment of firing clusters, reporting increases in detected cluster density of ≈30% in rat ventricular myocytes^4^. In our “low cluster density” cases, distributions of clusters were similarly generated based on plausible locations of RyR clusters but with an additional constraint of a minimum spacing of 1 μm between all clusters within a given z-disk. The number of clusters in this case was also reduced to comply with this constraint, with the total number of clusters 41% of those of the high density case. This was chosen as representative of a lower bound on cluster recruitment (as might occur with high pacing frequency and a negative amplitude-frequency relationship) but also revealed the impact of a 1 μm minimum cluster spacing requirement on detection, as suggested by Tian et al. (2017).

#### 3.2.2. Practical Implications

Our results suggest that CaCLEAN correctly detects the majority of Ca^2+^ release sites, with approximately one miss and one false positive out of every four or five sites (depending on site density). In our analysis, site spacing had a significant impact on both detection performance and the admissible window size associated with optimal performance. At the peak f1-score for cases including mitochondria acting as diffusion barriers, detection recall and precision were both 0.82 at 610 nm in the low cluster density case vs. 0.68 and 0.70 at 290 nm in the high cluster density case. In contrast, for the high cluster density results evaluated at the low cluster density peak f1-score admissible window of 610 nm, recall ≈0.43 thus indicating more false negative misses than true positive detection hits.

For useful detection performance, it is therefore important to consider the likely density of the events being detected in order to determine how far from the imaging plane such events are likely located. Those interested in using CaCLEAN to reconstruct three-dimensional maps of clusters should also be aware of how far from the imaging plane detected clusters are likely to be when choosing a slicing depth for reconstruction. In this case, the choice of slicing depth should be approximately twice the optimum performance admissible window. For example, in a case with high cluster density where precision and recall are equally important, a spacing of 580 nm would be recommended based on the values reported in Table 1.

#### 3.2.3. Confocal Fluorescence Imaging Resolution Limits the Impact of Subcellular Structures on Release Site Detection

The presence of heterogeneous diffusion (in the form of mitochondrial obstacles) in the investigated models was found to have a marginal but consistent negative impact on both recall and precision in CaCLEAN. While mitochondrial diffusion barriers introduced some additional false positive events, localized increases from Ca^2+^ reflecting against these obstacles did not significantly impair overall detection performance. Resolution limits may actually help to mitigate this factor, as such local increases in [Ca^2+^] at mitochondria-cytosol boundaries may be offset by lower values within the mitochondrial regions during interpolation into images. We hypothesize that structural heterogeneity due to the presence of subcellular structures (e.g., mitochondria, nucleus, transverse tubules, z-disks, etc.) is therefore unlikely to have a major impact on detection of point sources using CaCLEAN or related algorithms under imaging resolution conditions similar to those simulated here. Subsequent models similarly focused on Ca^2+^ release site detection performance might therefore benefit from significantly simplifying the computational domain by avoiding distinguishing mitochondrial regions.

### 3.3. Dependence of Findings on Modeling Assumptions

We evaluated the performance of a published algorithm for extracting calcium release site distributions from live confocal images using ground truth calcium release data that was generated using a spatially detailed model of a rat left ventricular cardiomyocyte. Several simplifying assumptions were made to create and analyse the model with this application in mind. Here we discuss these assumptions and also highlight ways in which the model could be extended and applied beyond the current study.

#### 3.3.1. Cellular Anatomy and Physiology

The modeled eight sarcomere domain was created by extruding a two-dimensional image from an electron tomography image stack. It therefore does not capture subtle structural variations along the longitudinal direction and assumes such changes are minimal over the relatively short (8 sarcomere, 16 μm) section modeled. Furthermore, sarcomere length in our model was fixed to 2 μm and we did not examine the effect of sarcomere length on detection results, as may occur in disease states (e.g., sarcomere lengths have been reported to shorten from 1.84 to 1.79 μm in a mouse model of lipotoxic diabetic cardiomyopathy (Flagg et al., 2009). However, we do not expect this change to affect our conclusions regarding RyR cluster detectability. In such cases, we still expect the key parameter affecting cluster detectability to be the RyR cluster density within a z-disk.

Our model simulated the rising phase (first 30 ms) of the calcium transient that the detection algorithm (CaCLEAN) is designed to operate over (Tian et al., 2017). RyR cluster behavior in the rising phase of the Ca^2+^ transient is known to be regulated by several signaling pathways which may be further complicated in disease and altered physiological states. While outside the scope of the current work, future model extensions might explore the impact of such pathways in the rising phase. For example, prolonged β-adrenergic stimulation can activate Ca^2+^/calmodulin–dependent kinase II (Maier and Bers, 2007; Camors and Valdivia, 2014) and nitric oxide signaling (Irie et al., 2015) to modulate RyR activity (Dries et al., 2016, 2018).

We chose to use a fixed Ca^2+^ release approach for the present study, since our goal was the evaluation of release site detection in simulated images rather than recapitulating inter-cluster feedback dynamics. This approach allowed greater control over the number of simulated releasing clusters in the model permutations investigated. Cases with mitochondrial diffusion barriers reduced the cytosolic volume by removing these regions, resulting in higher [Ca^2+^] throughout the cytosol. High cluster density permutations also increased global cytosolic [Ca^2+^]. These cases would therefore result in additional cluster activation from a CICR model compared to the low cluster density and homogeneously diffusing cases. We therefore found a fixed Ca^2+^ release model more appropriate for the control of this study given our focus. However, we acknowledge these mechanisms are important considerations for more general models seeking to explore inter-cluster Ca^2+^ signaling.

#### 3.3.2. Additional Ca^2+^ Transport Channels and Behavior

Ca^2+^ release in our model focused on sparks produced by active clusters of RyRs, the primary propagator of Ca^2+^ signals in healthy cardiomyocytes (Cheng et al., 1993). Active RyR clusters were implicitly considered to be triggered by L-type Ca^2+^ channels in the model. However, other channels involved in Ca^2+^ cycling that are more active in pathological states or during the decay phase could still have subtle influences on the Ca^2+^ signal and, therefore, potentially on release site detection. In particular, we did not define separate active populations of inositol triphosphate receptors (IP3Rs) as it is their Ca^2+^ release “puffs” are difficult to discern from RyR sparks based on signal shape and is further complicated by cross-talk (Harzheim et al., 2009; Wullschleger et al., 2017). During β-adrenoceptor activation, two-pore channels (TPCs) may also sensitize or induce spontaneous release of Ca^2+^ via the RyR (Capel et al., 2015). Ca^2+^ entry via the reverse mode of the sodium-calcium exchanger (NCX) has also been reported during the rising phase of the Ca^2+^ transient (Bers, 2002). It is possible that cytosolic Ca^2+^ increases due to IP3R dependent puffs or reverse mode NCX may contribute to additional false positive detection events in addition to RyR sensitization. However, there remains uncertainty regarding the spatial organization and quantifiable contribution of these low amplitude events (Harzheim et al., 2009; Horn et al., 2013; Eisner et al., 2017).

Sarco/endoplasmic reticulum Ca^2+^-ATPase (SERCA) re-uptakes Ca^2+^ from the cytosol into intracellular stores and has been shown to colocalize with RyRs at z-disks (Drago et al., 1998; Eisner et al., 2017; Hadipour-Lakmehsari et al., 2019). SERCA (or effluxing NCX) located near a releasing RyR cluster might alter Ca^2+^ diffusion into the imaging plane by reducing signal amplitude or symmetry (depending on the orientation of these channels in relation to the imaging plane). While we have previously reported that SERCA flux is most prominent after the Ca^2+^ transient peak (Rajagopal et al., 2015), SERCA activity is non-zero during the Ca^2+^ upstroke and has been shown to modulate release (Maxwell and Blatter, 2017). This could hypothetically impact detection using CaCLEAN, which assumes symmetric point sources when deconvolving the fluorescence signal.

#### 3.3.3. Relevance to Other Cell Types

Our model was based on experimental data from healthy male adult Wistar rat ventricular myocytes. We hypothesize our findings may extend to other cell types where RyRs are equally well organized (e.g., Purkinje, Hirose et al., 2008 and atrial, Bootman et al., 2006 cells). Users primarily interested in how Ca^2+^ release site detectability changes across species, under disease conditions, or at different stages of development may wish to consider repeating our approach under these conditions. While our model focused on a rising phase Ca^2+^ transient of 30 ms, experimental application of CaCLEAN (and its evaluation with our modeling approach) should still be applicable to cases with longer or shorter pre-contractile periods within confocal temporal imaging resolution. Based on our findings here, we hypothesize that detection performance will still be dependent on release site distance from the imaging plane and inter-site spacing.

### 3.4. *In silico* Models Can Reveal Variables Unobtainable *in vitro* or *in vivo*

The results presented in this work (particularly Figures 5–7, Table 1) are provided as a reference tool for users interested in experimentally detecting Ca^2+^ release sites from live cardiomyocytes using CaCLEAN. These findings and the approach developed here to quantify detection performance may also extend more broadly to other algorithms seeking to accurately detect three-dimensional distributions of point sources from two-dimensional images. Users interested in Ca^2+^ release site detection with substantially different experimental configurations (e.g., spatial and temporal imaging resolution, optical PSF, or expected noise) can modify the provided Python script(s) (see Data Availability below) with parameters to their specifications to tune detection performance assessment for their application.

The computational model results include the Ca^2+^ and fluorescence-bound Ca^2+^ data discussed here, along with solved fields for unbound fluorophore, calmodulin, Ca^2+^-bound calmodulin, ATP, Ca^2+^-bound ATP, and troponin C. These results may be useful to others interested in a high-resolution representation of the mechanics of these fields during the rising phase of the Ca^2+^ signal transient in a rat ventricular cardiomyocyte. The capability to simulate imaging data from the model results also means it could serve as a testing and validation platform for other analysis tools operating on confocal fluorescence imaging of Ca^2+^ release in cardiomyocytes that would benefit from using the high resolution continuum model as reference. For instance, this could be used to further improve CaCLEAN or other approaches seeking to identify RyR clusters by serving as a training set for improved detection or segmentation. The code and supporting datasets for the model, confocal data simulator, and detection performance analysis are therefore freely available for subsequent research (see Data Availability below). Finally, this study highlights how computational methods may be used to combine diverse experimental data into a consistent physical framework to establish ground truth values that may not otherwise be experimentally available.

## 4. Materials and Methods

### 4.1. Finite Element Model of Reaction-Diffusion in an Eight Sarcomere Section of a Cardiomyocyte

The reaction-diffusion finite element (FE) model here builds on a previous FE model of a half-sarcomere (Rajagopal et al., 2015). From electron tomography (ET) images of healthy male adult Wistar rat ventricular myocytes, a three-dimensional axial region with a thickness of approximately 0.875 μm was segmented. This region represented approximately half of a single sarcomere, with the z-disk a plane through the center of the thickness of the domain. The region was approximately 11 μm in diameter, varying with the segmented surface.

From this half-sarcomere image stack, the central slice representing the level of the z-disk was extracted and regions representing myofibrils and mitochondria were manually segmented. This two-dimensional slice was then extruded 16 μm to create a three dimensional volume.

Two configurations were considered to assess the impact of mitochondria: one in which the interior of the modeled domain is a homogeneous material continuum and one in which the regions representing mitochondria were subtracted from the myofibrillar and cytoplasmic domain. The latter case was based on the assumption that the calcium buffering activity of mitochondria is negligible.

#### 4.1.1. Definition of RyR Cluster Distributions

Ryanodine receptor (RyR) locations were defined algorithmically, using a spatial statistics method based on nearest neighbor distances of experimentally-derived RyR locations (Rajagopal et al., 2015). These distributions were determined from confocal images of left ventricular cardiomyocytes of a healthy adult male Wistar rat using multiple passes of a band-pass filter detector, following the technique described by Soeller and Cannell (2002). A non-parametric approach fitted nearest-neighborhood distributions within admissible locations for clusters to the distributions acquired from the confocal imaging-based protein localization data. The admissible locations of RyR clusters were regions tracing the borders of myofibrils, mitochondria, and the sarcolemma (i.e., RyR clusters were not placed inside organelles). Each z-disk in this model contains unique cluster location distributions.

To evaluate the influence of spacing on the RyR cluster detection performance of CaCLEAN, two sets of constraints on the RyR distributions were considered: (1) RyR clusters were assigned with locations based directly on statistical analysis of experimental data and with the total number of RyR clusters *N* = 123 per z-disk (984 total); (2) a minimal distance constraint of 1 μm between RyR cluster centers was enforced and the total number of clusters was reduced to *N* = 51 per z-disk (408 total) to allow for this constraint. Eight unique cluster distributions were generated for each case and were spaced 2 μm apart along the extruded axis model volume to represent z-disk RyR populations. Linear tetrahedral meshes were constructed on these domains, consisting of: (1) 1,436,943 nodes, 8,222,684 elements in the high cluster density case; and (2) 1,318,942 nodes, 7,504,655 elements in the low cluster density case. These meshes included increased refinement in the regions containing and surrounding the modeled RyR locations.

Spherical regions 100 nm in radius were defined around nodes nearest to the determined location for each RyR cluster. Nodes lying within this sphere were prescribed density amplitudes exponentially decreasing as a function of the square of their radial position from the central node. RyR cluster release times were sampled from an exponential distribution with a characteristic decay constant of 6.7 ms, following the findings of Wang et al. (2001).

#### 4.1.2. Modeling RyR Cluster Ca^2+^ Release

In our previous model (Rajagopal et al., 2015) two approaches for simulating the release of Ca^2+^ through clusters of RyRs were considered: (1) by specifying release amplitudes and firing times using a mathematical equation composed of an exponential rise and decay; and (2) by enabling clusters of RyRs to operate via CICR sensitivity using a deterministic approximation of stochastic properties of clusters of RyRs. While the CICR model enabled the representation of inter-cluster feedback triggering, these cases were rare and did not significantly impact the overall spatiotemporal profile of cytosolic calcium. This is because the simulations represented the scenario that Ca^2+^ through voltage-gated L-type calcium release channels on the t-tubules triggered the calcium release through each RyR cluster. Therefore, local [Ca^2+^] in the model was more dependent on placement of possible release sites than the representation of the CICR mechanism. Indeed, we show in our previous study that the spatiotemporal pattern of rise in cytosolic Ca^2+^ due to CICR induced by L-type Ca^2+^ is very similar to that generated by the phenomenological model of RyR cluster Ca^2+^ release. Based on this, we used the phenomenological fixed release approach in the present model.

We used an effective diffusion coefficient for diffusion of Ca^2+^ released in the cytosol. This diffusion is coupled with reaction terms that mediate Ca^2+^ binding and unbinding to buffers within the cytosol. Further details on the reaction-diffusion equations for the buffers modeled and the ordinary differential equation model describing release of Ca^2+^ from RyR clusters have been previously reported (Rajagopal et al., 2015). We set stable initial conditions for the buffers examined as previously reported (Rajagopal et al., 2015) rather than establishing stability over several cycles. The RyR clusters modeled as active during the simulations were considered fully recovered prior to excitation. In our deterministic model, all buffers, calcium and buffer-bound calcium concentrations are set to represent chemical equilibrium at rest until Ca^2+^ is released from the RyR cluster sources. The initial conditions and parameters used have also been previously reported (Rajagopal et al., 2015) and were based on previously published values (Izu et al., 2006; Picht Eckard et al., 2011). Numerical solution of the resulting system of partial differential equations was accomplished using the OpenCMISS-Iron library (Bradley et al., 2011). The ordinary differential equations describing the release of Ca^2+^ from RyR source clusters were defined using CellML (Garny et al., 2008) and coupled to the FE model as source terms (Nickerson et al., 2015).

### 4.2. Simulation of Confocal Fluorescence Signals From FE Model Results

To simulate confocal fluorescence results, the irregularly distributed node-based fluorescence-bound Ca^2+^ field from the FE model was first interpolated onto a regular grid at a resolution of 53.75 nm in each direction. The data were temporally sampled at 5 ms intervals over the simulated time period of 30 ms, producing simulated imaging data for 7 timesteps. Nodal positions within mitochondria in the “with mitochondria” permutations were ascribed the initial and background value FCa = 2.08. Discrete natural neighbor (Sibson) interpolation (Park et al., 2006) was chosen on the basis that it can be used to generate regularly-spaced three-dimensional data, scales well for large datasets, and does not require additional parameterization. The implementation used was version 1.7 of the naturalneighbor python package, available from the Python Package Index under the MIT license.

A point spread function (PSF) was generated as a normalized function of a multivariate Gaussian distribution applied in three dimensions (see Figure 2B). These distributions had a full width at half maximum (FWHM) of 410 nm in x and y and 1,800 nm in z (where the z-axis represents the through-imaging-plane direction, shown as “y” in Figure 2B). These values were based on reported estimates of the dimensions of a PSF from a Visitech confocal microscope (Plumb et al., 2015). The resolution of the PSF image was chosen to be the same as the interpolated model data (53.75 nm in each direction).

The interpolated model imaging data was then convolved with the PSF in three dimensions using the SciPy convolve algorithm from the signal processing module. The resulting grid was then downsampled to a pixel resolution of 215 nm, following the resolution of the original CaCLEAN paper (Tian et al., 2017). Light noise (SNR = 100) was also added to the image data. Representative slices were sampled along the y-axis of the FE model, producing 22 two-dimensional simulated microscopy images for each of the four reported model permutations.

### 4.3. Application of CaCLEAN to Simulated Fluorescence Data

The CaCLEAN algorithm was obtained from the author's GitHub repository: https://github.com/qhtian/CaCLEAN. The Matlab-based scripts were run using Matlab version R2017b. The function CICRcleanSimp was used to generate the CaCLEAN release map and the function CRUProps was used to segment the release map into individual calcium release units (CRUs).

### 4.4. Classifying and Quantifying Ca^2+^ Release Site Detection Performance

A statistical classification approach was used to assess the performance of RyR cluster detection. Modeled cluster centers within the admissible window were considered the actual/ground truth class: TP (ground truth). Detection results were considered the predicted class. Detected RyR cluster sites were defined as determined by the CaCLEAN CRUProps function, which segments cluster regions using Matlab's built-in watershed algorithm and identifies centroids of segmented regions.

For each modeled cluster location, a TP (detected) classification was assigned if a TP (ground truth) cluster center lied within an available segmented CaCLEAN cluster region (or within a 1 pixel tolerance). When more than one TP (ground truth) fell within a CaCLEAN-detected cluster region, the detected cluster with the nearest centroid to the TP (ground truth) location was marked TP (detected). After classification as TP (detected), the associated CaCLEAN-detected site would be removed from the list of available matches. After iterating through the TP (ground truth) clusters, remaining TP (ground truth) unmatched with detected clusters were classified as false negatives (FN). Remaining CaCLEAN-detected sites unmatched with TP (ground truth) were classified as false positives (FP). Note that in this case

(1) $$\begin{array}{r} \text{TP }(\text{ground truth})=\text{TP }(\text{detected})+\text{FN}. \end{array}$$

Three statistical binary classification performance measures were considered: recall, precision and f1-score (Nisbet et al., 2018; Berrar, 2019). Recall (also known as sensitivity, hit rate, or true positive rate) was defined such that

(2) $$\begin{array}{r} \text{Recall}=\frac{\text{TP }(\text{detected})}{\text{TP }(\text{ground truth})}. \end{array}$$

Recall therefore gives the fraction of actual modeled clusters within an admissible window that were correctly detected by CaCLEAN. Precision (also known as positive predictive value) was defined such that

(3) $$\begin{array}{r} \text{Precision}=\frac{\text{TP }(\text{detected})}{\text{TP }(\text{ground truth})+\text{FP }(\text{detected})}. \end{array}$$

Precision therefore identifies the fraction of the detected clusters within an admissible window that were correct (not false positives). Another useful parameter indicating the combined effect of both precision and recall is the f1-score (also known as f-measure or f-score), which measures the harmonic mean of these two variables i.e.,

(4) $$\begin{array}{r} \text{F}1-\text{Score}=\frac{2}{\frac{1}{\text{Recall}}+\frac{1}{\text{Precision}}}. \end{array}$$

This provides a single combined performance metric that equally weights the impact of false positive and false negative events. In all three performance metrics, higher values indicate better performance.

The above definition of recall may be considered “cumulative recall” in our application, identifying the detection fraction of all clusters within a given admissible window. To determine the detection fraction of clusters at a given z distance from the simulated imaging plane, we also defined an alternative “differential recall” such that

(5) $$\begin{array}{r} \text{Differential Recall}=\frac{d(\text{TP }(\text{detected}))}{d(\text{TP }(\text{ground truth}))}. \end{array}$$

This measured the fraction of modeled clusters detected by CaCLEAN in 10 nm spaced bands above and below the imaging plane. Only bands with at least one TP (ground truth) were considered. Mean values for this detection fraction were acquired over the 22 simulated imaging planes. A Savitzky-Golay filter (polynomial order 3, frame length 21) was then applied to smooth the results as shown in Figure S5. Single-term Gaussian fits were also applied to identify trends in the resulting curves, as shown in Figure 7.

## Data Availability Statement

The code used to solve the FE model is available in the repository: https://github.com/uomsystemsbiology/CardiacCalcium_FiniteElement. The code used to simulate the microscopy images and evaluate detection is available in the repository: https://github.com/uomsystemsbiology/CardiacCalcium_TestCaCLEAN. Supporting datasets may also be downloaded from the project collection hosted on figshare: https://doi.org/10.26188/5cd1286834075.

## Author Contributions

DL developed, analyzed, and curated the computational model. DL, CS, and VR contributed to the initial conceptualization of this study. CS, EC, and VR supervised the project. EC and VR directed administration of the project and funding acquisition. DL, AT, HR, CS, EC, and VR contributed to the development of the methodology and preparation of original and final drafts.

### Conflict of Interest

The authors declare that the research was conducted in the absence of any commercial or financial relationships that could be construed as a potential conflict of interest. The reviewer MAC declared a past collaboration with one of the authors CS.

## Footnotes

^1^Where the two-dimensional simulated image plane represented x-y axes.

^2^True negatives were not considered in this application, as they would represent the set of all remaining locations that were neither modeled nor detected.

^3^Immuno-labeled confocal data populations may still slightly underestimate cluster density owing to diffraction-limited resolution.

^4^See Figure 3Cb in Tian et al. (2017).
