# Supplementary material for: Assessing Cardiomyocyte Excitation-Contraction Coupling Site Detection From Live Cell Imaging Using a Structurally-Realistic Computational Model of Calcium Release
Source: Front Physiol. 2019 Oct 2;10:1263. doi: 10.3389/fphys.2019.01263 (PMC6783691; doi:10.3389/fphys.2019.01263)
Supplement: Supplementary file 1 [file Data_Sheet_1.pdf]

Supplementary Material

0.1 Function fits for precision and recall data

Four-term Gaussian fits were applied to the mean precision and recall values from Figure S2 to attain continuous functions i.e.,

$$\text{Precision, Recall} = a1\left(\exp\left(-\frac{(w_a - b1)^2}{c1^2}\right)\right) + a2\left(\exp\left(-\frac{(w_a - b2)^2}{c2^2}\right)\right) + a3\left(\exp\left(-\frac{(w_a - b3)^2}{c3^2}\right)\right) + a4\left(\exp\left(-\frac{(w_a - b4)^2}{c4^2}\right)\right), \quad \text{(S1)}$$

where w_a represents the admissible window parameter. Parameters $a1 ... c4$ are defined for each modeled case for recall in Table S1 and for precision in Table S2. Illustrations of these curve fits applied to the mean value data can be found in Figure S3. These curves can be used to estimate performance values as a function of the admissible window- rather than users being limited to the values reported in Table 1 and Table 2, or digitising the graphs.

1 SUPPLEMENTARY TABLES AND FIGURES

1.1 Tables

Table S1. Parameters for four-term Gaussian fit of mean **recall** values (to be applied to Equation S1)

Case	a1	b1	c1	a2	b2	c2	a3	b3	c3	a4	b4	c4
1	0.3644	34.61	303.8	-1.147e+05	-9539	2483	0.06817	466.6	258.5	1.545e+06	-3.959e+04	1.029e+04
2	0.03391	155	74.71	0.7532	-271.5	687.3	0	390.9	0.1121	0.2895	317.9	1825
3	-0.01641	1265	225.8	0.0928	473.7	343.5	0.007959	619.3	23.27	1.005	-676.4	2402
4	0.2007	390.4	530.6	0.01895	602.2	78.54	-0.0118	635.9	92.04	0.8585	-939.6	2875

Table S2. Parameters for four-term Gaussian fit of mean **precision** values (to be applied to Equation S1)

Case	a1	b1	c1	a2	b2	c2	a3	b3	c3	a4	b4	c4
1	0.9511	1561	645.9	0.4739	501	282.4	0.5692	856.5	407	0.3745	252.6	198.3
2	0.9003	1521	676.3	0.4136	459.6	281.4	0.2783	239.9	182.4	0.5211	805.7	420.7
3	0.9447	1558	637.1	0.3049	572.5	258.8	0.2563	301.9	221.6	0.5595	874.8	407.7
4	0.9637	1461	693.1	-0.00177	759.3	17.99	0.2079	365	256	0.5405	716.3	417

1.2 Figures

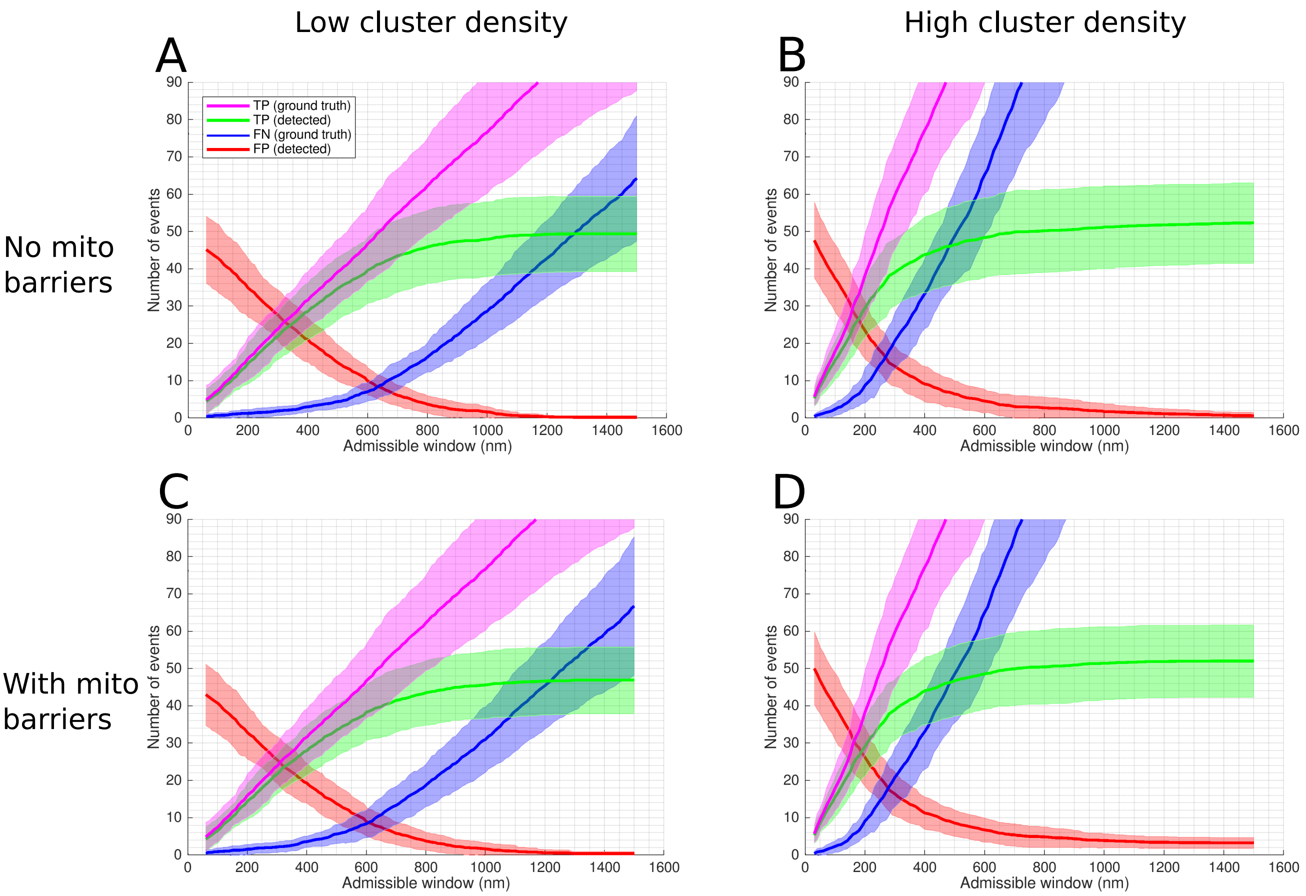

Figure S1. Statistical classifiers of CaCLEAN performance on simulated microscopy data in four model permutations. Statistical classifiers for CaCLEAN results shown as a function of the size of the admissible window for each of the four model permutations. In each case, 22 images were simulated with equidistant spacing through the model volume. Shaded regions indicate one standard deviation of values within the group of 22 image slices. (A) A minimum spacing of 1 μm is enforced between modeled clusters, no mitochondria present in the volume (resulting in a continuous domain across the mitochondrial regions). (B) Cluster spacing is determined by statistical analysis of RyR cluster distributions from immuno-labelled super-resolution microscopy data, no mitochondria present in the volume. (C) A minimum cluster spacing of 1 μm is enforced between modeled clusters, mitochondrial regions are subtracted from the volume, acting as barriers to diffusion. (D) Cluster spacing is determined by statistical analysis of RyR cluster distributions from immuno-labelled super-resolution microscopy data, mitochondrial regions are subtracted from the volume, acting as barriers to diffusion.

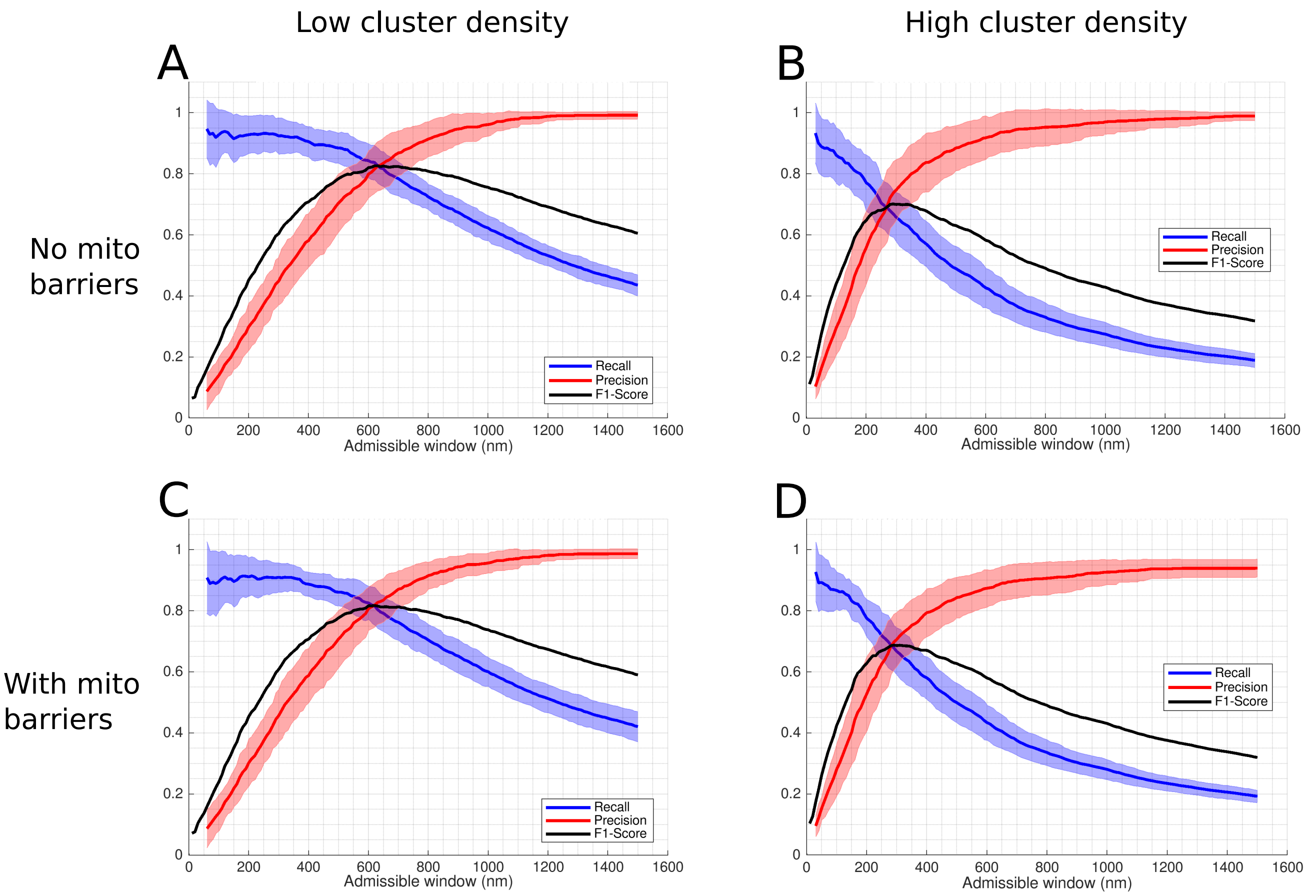

Figure S2. Recall, precision, and f1-score for all model permutations Recall, precision and f1-score evaluated based on classification results. Shaded regions indicate one standard deviation of recall and precision values. The f1-score shown is evaluated based on the mean values for precision and recall (solid red and blue lines). (A) A minimum spacing of 1 μm is enforced between modeled clusters, no mitochondria present in the volume (resulting in a continuous domain across the mitochondrial regions). (B) Cluster spacing is determined by statistical analysis of RyR cluster distributions from immuno-labelled super-resolution microscopy data, no mitochondria present in the volume. (C) A minimum cluster spacing of 1 μm is enforced between modeled clusters, mitochondrial regions are subtracted from the volume, acting as barriers to diffusion. (D) Cluster spacing is determined by statistical analysis of RyR cluster distributions from immuno-labelled super-resolution microscopy data, mitochondrial regions are subtracted from the volume, acting as barriers to diffusion.

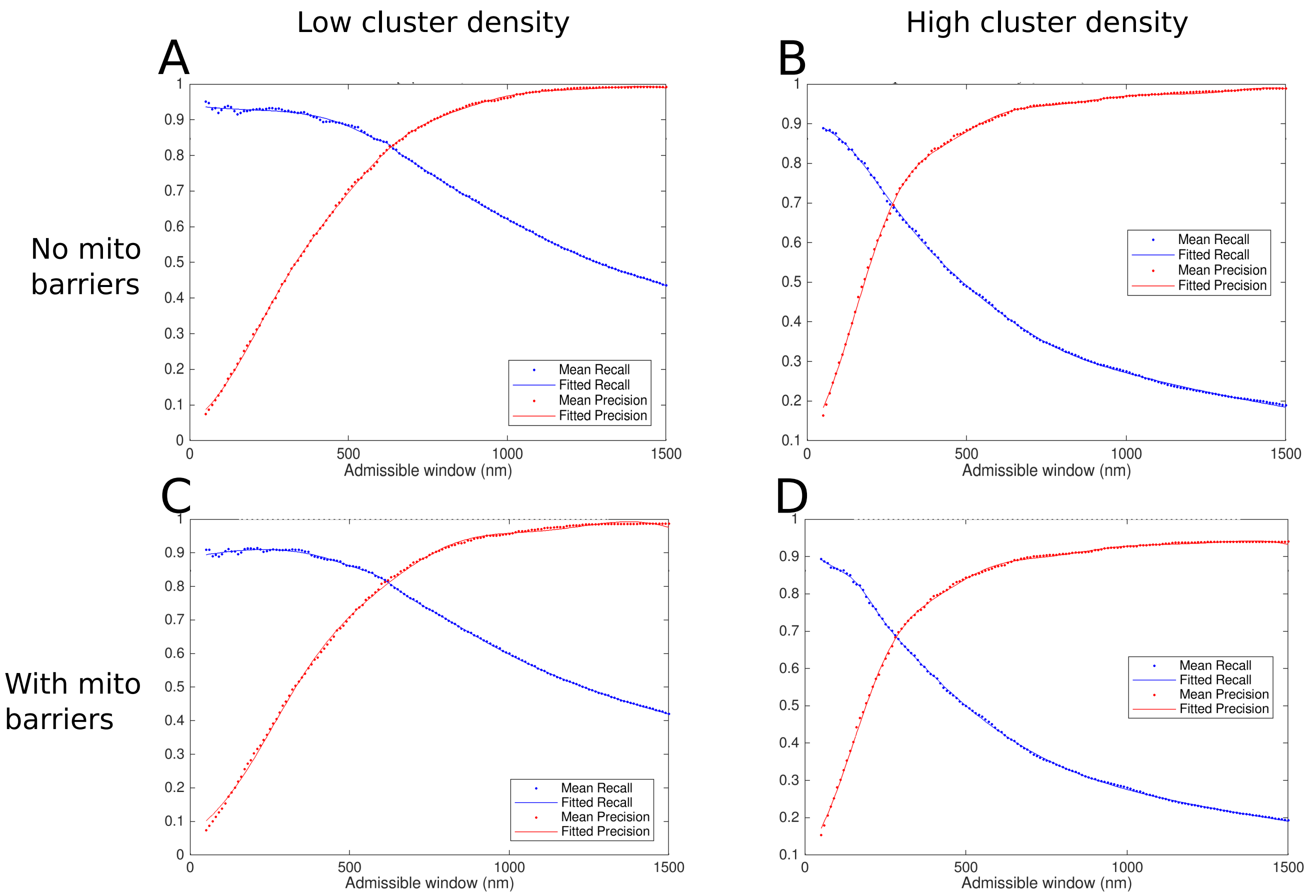

Figure S3. Four-term Gaussian curves fit to mean recall and precision values Four-term Gaussian functions were fit to the mean (solid line) values shown in Figure S2. This allows for the evaluation of precision and recall values as a function of admissible window.

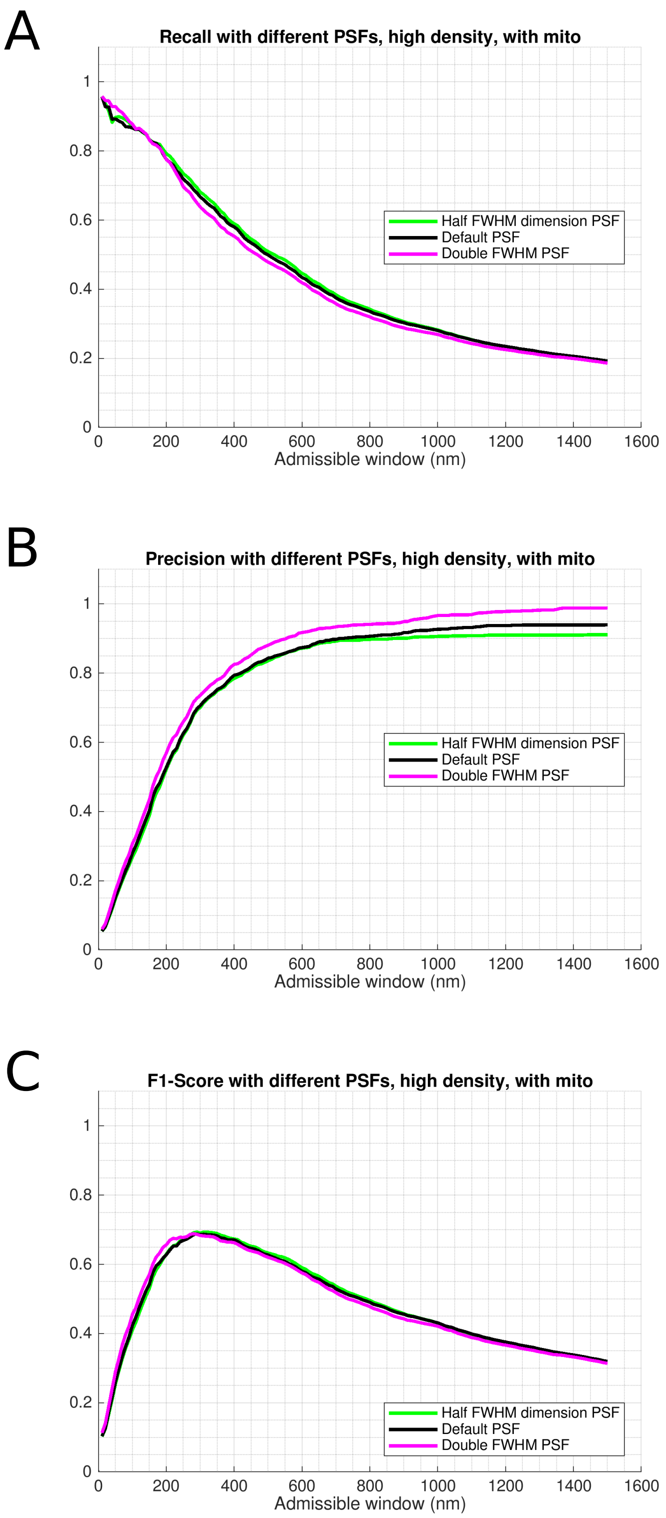

Figure S4. Recall, precision, and f1-score with alternative PSFs. Performance metrics shown for the high cluster density, with mitochondria case (Figure 5B), with two alternate point spread functions (PSFs) shown in comparison to the default. The default PSF (black) full width at half maximum (FWHM) dimensions were 410 nm in the image x-y plane and 1800 nm in the through-image z plane were based on published values using a Visitech confocal microscope (Plumb et al., 2015). The half FWHM PSF (green) was case FWHM dimensions were 205 nm in xy, 900 nm in z. The double FWHM PSF (magenta) case FWHM dimensions were 820 nm in xy, 3600 nm in z. Plots show (A) recall, (B) precision, and (C) f1-score performance results using microscopy images simulated with the three PSFs.

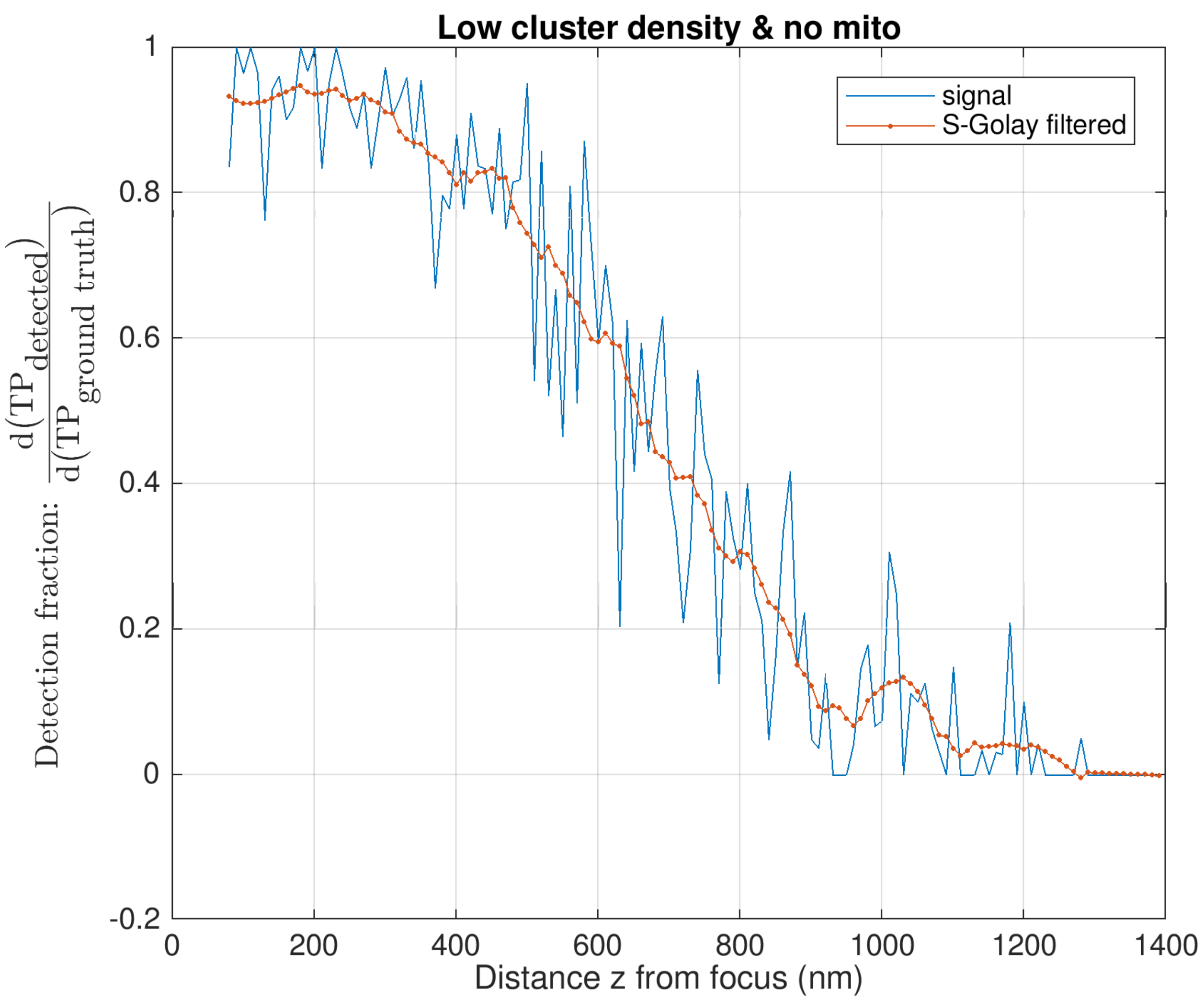

Figure S5. Smoothing filter applied to differential recall data. The differential recall results (shown here in blue) were smoothed using a Savitzky-Golay filter (polynomial order 3, frame length 21, results shown in red). The example data shown here is from the low density, no mitochondria case, with the filtered results here corresponding with the magenta data points in Figure 7.
